# Identification of senescence rejuvenation mechanism of *Magnolia officinalis* extract including honokiol as a core ingredient

**DOI:** 10.18632/aging.206207

**Published:** 2025-02-21

**Authors:** Yun Haeng Lee, Eun Young Jeong, Ye Hyang Kim, Ji Ho Park, Jee Hee Yoon, Yoo Jin Lee, So Hun Lee, Yeon Kyung Nam, So Yoon Cha, Jin Seong Park, So Yeon Kim, Youngjoo Byun, Song Seok Shin, Joon Tae Park

**Affiliations:** 1 Division of Life Sciences, College of Life Sciences and Bioengineering, Incheon National University, Incheon 22012, Republic of Korea; 2 Life Sciences R&D Center, Hyundai Bioland co. ltd, 22, Osongsaengmyeong 2–ro, Osong–eup, Heungdeok–gu, Cheongju–si, Chungcheongbuk–do, Republic of Korea; 3 College of Pharmacy, Korea University, Sejong 30019, Republic of Korea; 4 Convergence Research Center for Insect Vectors, Incheon National University, Incheon 22012, Republic of Korea

**Keywords:** mitochondria, ROS, aging rejuvenation, magnolia officinalis, honokiol

## Abstract

Reactive oxygen species (ROS) contribute to aging by mainly damaging cellular organelles and DNA. Although strategies to reduce ROS production have been proposed as important components of anti-aging therapy, effective mechanisms to lower ROS levels have not yet been identified. Here, we screened natural compounds frequently used as cosmetic ingredients to find substances that reduce ROS levels. *Magnolia officinalis* (*M. officinalis*) extract significantly lowered the levels of ROS in senescent fibroblasts. A novel mechanism by which *M. officinalis* extract restores mitochondrial function to reduce ROS, a byproduct of inefficient electron transport, was discovered. The reduction of ROS by *M. officinalis* extracts reversed senescence–associated phenotypes and skin aging. Then, honokiol was demonstrated as a core ingredient of *M. officinalis* extract that exhibits antioxidant effects. Honokiol functions as an oxygen radical scavenger through redox processes, also significantly reduced ROS levels by restoring mitochondrial function. In summary, our study identified a novel mechanism by which *M. officinalis* extract reverses aging and skin aging by reducing ROS through restoring mitochondrial function. These new findings will not only expand our understanding of aging and associated diseases, but also provide new approaches to anti–aging treatments.

## INTRODUCTION

Alterations in the composition and function of organelles, particularly the degeneration of mitochondria, are among the major hallmarks of senescence [1, 2]. As senescence progresses, structural changes occur in our mitochondria, such as an increase in their size and mass, which ultimately leads to functional defects [3]. Defective mitochondria create reactive oxygen species (ROS) as byproducts through leaking electrons from the electron transport complex (ETC) [4]. Defective mitochondria serve as both a source of mitochondrial ROS generation and a target of mitochondrial ROS–induced oxidative stress [5]. Increased oxidative stress further damages mitochondria, subsequently increasing mitochondrial ROS generation [5]. Excessive level of ROS impairs the structure and function of other organelles, consequently leading to senescence [6]. Previous studies conducted on mice that are lacking in superoxide dismutase 1 (SOD1), an enzyme that destroys free radicals in the mitochondrial intermembrane space and matrix, have verified this causal relationship [7]. SOD1 deficiency leads to premature aging in skeletal muscle due to increased superoxide anions (O_2_^●−^) generation and subsequent oxidative damage [7]. Thus, methods to decrease mitochondrial oxidative stress could be advantageous as an anti–aging treatment [8, 9]. The usefulness of this approach is further supported by the finding that the mitochondrial–specific antioxidant, MitoQ, reduces mitochondrial ROS generation and reverses neurodegeneration [10]. Given the role of ROS-induced oxidative stress on senescence, better understanding the mechanisms that control it is an urgent priority.

The skin is composed of the epidermis, made of mainly epithelial tissue, and the dermis, made of mainly connective tissue [11]. Collagen, which accounts for 90% of the dermis layer, makes skin tissue elastic and maintains the shape and elasticity of the skin [11]. The most obvious phenomenon in skin aging is the decrease in collagen, which causes a reduction in skin elasticity and a formation of wrinkles [12]. In particular, ROS has been suggested as the main cause of skin aging by stimulating collagen oxidation and collagen–elastin chain cleavage [12]. Therefore, strategies using antioxidants that neutralize free radicals are used to inhibit skin aging [13]. For instance, vitamin C, a well–known antioxidant, mitigates the degradation of endogenous collagen in skin tissue by reducing the production of free radicals [14]. Additionally, the antioxidant niacinamide reduces skin damage by inhibiting collagen damage caused by free radicals [15]. Therefore, antioxidant–based techniques may be useful treatments for slowing or stopping skin aging.

*Magnolia officinalis* (*M. officinalis*) is an evergreen tree distributed in East Asia, mainly found in Korea, Japan, and China [16]. *M. officinalis* is a medicinal plant historically utilized in traditional medicine for its anti–inflammatory, antibacterial, and antioxidant properties [17]. Moreover, *M. officinalis* extract has been utilized as an efficacious cosmetic material to enhance skin whitening and diminish skin pigmentation [18]. However, the aging rejuvenation mechanism and core ingredient of the aging rejuvenation effect of *M. officinalis* extract have not yet been discovered. Consequently, comprehending the mechanism of the aging rejuvenation effect of *M. officinalis* extracts and identifying the core ingredients will further expand the application fields of *M. officinalis* extracts as aging rejuvenation agents and cosmetic materials.

Mitochondria are the main organelles that generate free radicals that cause cell damage [19]. Mitochondria consume over 90% of oxygen, and complexes I and III of the ETC convert 1% to 5% of oxygen into ROS [19]. The development of various mitochondrial-specific dyes has made it possible to measure mitochondrial ROS, among which dihydrorhodamine 123 (DHR123) and MitoSOX are extensively used. DHR123 is a reduction product of rhodamine 123, a dye that passively diffuses across cell membranes and selectively stains mitochondria [20]. Thus, DHR123 reacts with ROS present in mitochondria and is oxidized to cationic rhodamine 123, allowing for measurement of ROS levels present in mitochondria [21]. MitoSOX has positively charged triphenylphosphonium ion (TPP^+^), which accumulates in the negatively charged mitochondrial matrix [22]. Dehydroethidium bound to TPP^+^ reacts with superoxide in the mitochondrial matrix [22].

Here, we aimed to find substances that effectively reduce mitochondrial ROS generation using a library of natural products widely used as cosmetic materials. *M. officinalis* extract was found to reduce ROS levels by restoring mitochondrial function. Furthermore, we found out which components of *M. officinalis* extract were responsible for these effects. Here, in order to ameliorate senescence-associated phenotypes and reverse skin aging, we propose a unique ROS-reducing mechanism of *M. officinalis* extract.

## RESULTS

### Mitochondrial ROS levels in senescent fibroblasts are significantly reduced by *M. officinalis* extract

**Figure 1:**
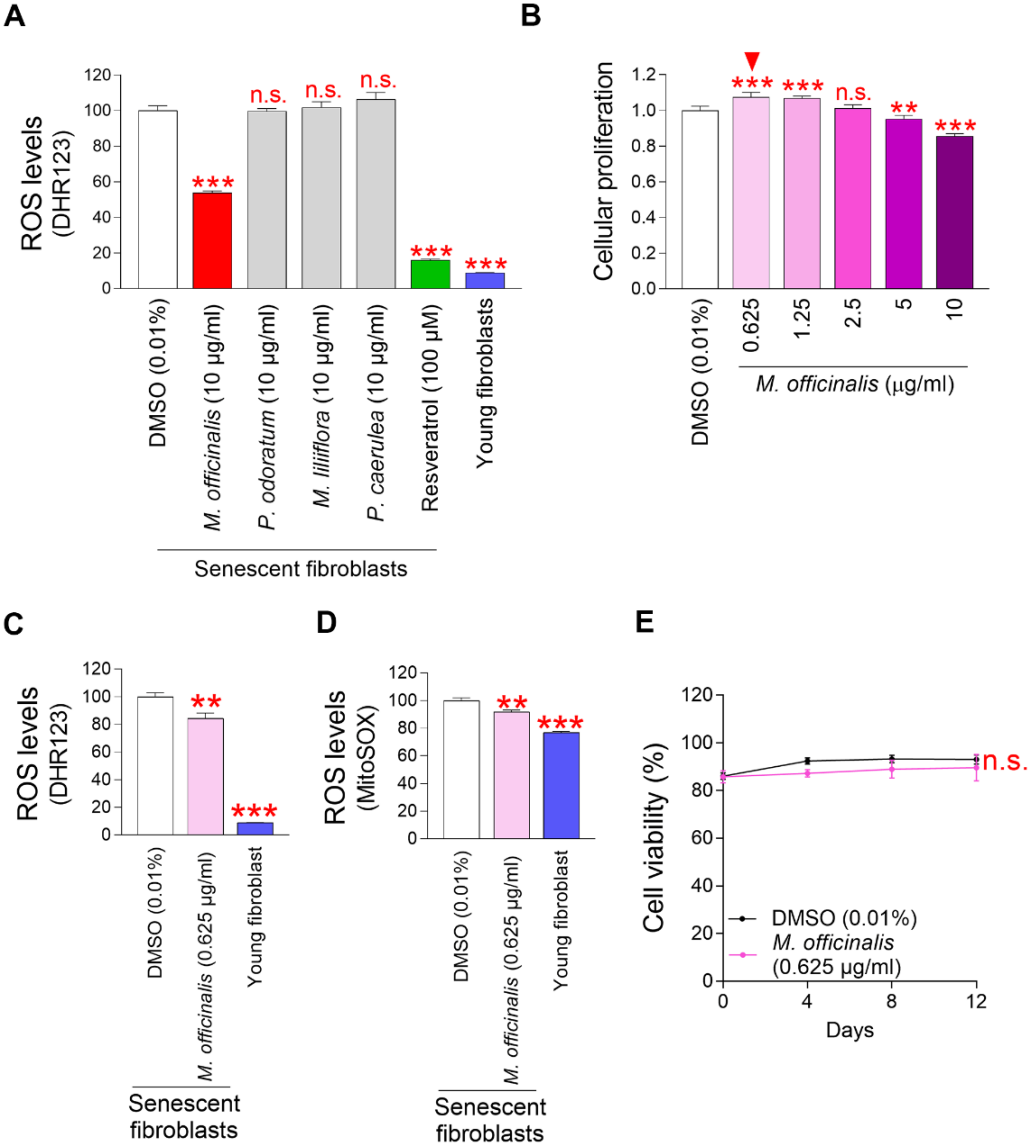
**Mitochondrial ROS levels in senescent fibroblasts are significantly reduced by *M. officinalis* extract.** (**A**) Senescent fibroblasts were treated with *M. officinalis*, *P. odoratum*, *M. liliiflora* and *P. caerulea* extract at a 10 μg/ml. On day 12, the impact on mitochondrial ROS levels was evaluated. DMSO was diluted in the medium to 0.01% to serve as DMSO control. Use of dihydrorhodamine 123 (DHR123) for flow cytometric analysis of mitochondrial ROS levels. Resveratrol (100 μM) and young fibroblasts were used as a positive control. n.s. (not significant), ***P < 0.001, Student's t–test. Mean ± S.D., N = 3. (**B**) On day 12 following treatment, cellular proliferation in senescent fibroblasts was assessed at varying doses of *M. officinalis* extract (0.625, 1.25, 2.5, 5, and 10 μg/ml). n.s. (not significant), **P < 0.01, ***P < 0.001, Student's t–test. Mean ± S.D., N = 3. Arrowhead shows the optimal concentration of *M. officinalis* extract for promoting cellular proliferation. (**C**) DMSO (0.01%) or *M. officinalis* extract (0.625 μg/ml) were administered to senescent fibroblasts for 12 days. Then, the flow cytometric analysis of ROS (DHR123) was conducted. Young fibroblasts were used as a positive control. **P < 0.01, ***P < 0.001, Student's t–test. Mean ± S.D., N = 3. (**D**) DMSO (0.01%) or *M. officinalis* extract (0.625 μg/ml) were administered to senescent fibroblasts for 12 days. Use of MitoSOX for flow cytometric analysis of mitochondrial ROS levels. Young fibroblasts were used as a positive control. **P < 0.01, ***P < 0.001, Student's t–test. Mean ± S.D., N = 3. (**E**) Senescent fibroblasts were treated with DMSO (0.01%) or *M. officinalis* extract (0.625 μg/ml). Measurement of cell viability after 0, 4, 8, and 12 days of treatment. n.s. (not significant), two–way ANOVA followed by Bonferroni’s post–hoc test. Mean ± S.D., N = 3.

Among the commonly used cosmetic materials, *M. officinalis*, *Polygonatum odoratum* (*P. odoratum*), *Magnolia liliiflora* (*M. liliiflora*), and *Passiflora caerulea* (*P. caerulea*) extracts were used to identify potential candidates that could significantly reduce mitochondrial ROS levels. *M. officinalis* extract is recognized as effective in skin hydration and soothing [17]. *P. odoratum* extract is effective for skin whitening and moisturizing [23]. *M. liliiflora* has been used since ancient times for its skin-relaxing properties [24]. *P. caerulea* extract is widely used as a cosmetic ingredient because it is rich in natural substances with antioxidant functions such as anthocyanins and polyphenols [25, 26]. Each extract was treated to senescent fibroblasts at a concentration of 10 μg/ml for a period of 12 days. Subsequently, we examined the impact of each extract on ROS levels by using DHR123 which can specifically detect ROS in mitochondria [20]. A strong antioxidant, resveratrol, served as a positive control [27]. Resveratrol significantly reduced ROS levels in senescent fibroblasts (Figure 1A). Young fibroblasts were also employed as a positive control. As expected, young fibroblasts had substantially lower ROS levels than senescent fibroblasts treated with DMSO [28] (Figure 1A). Among the four substances, *M. officinalis* extract significantly reduced ROS levels (Figure 1A).

However, *P. odoratum* extract, *M. liliiflora* extract, and *P. caerulea* extract, which are recognized for their efficacy in skin care, were not beneficial in lowering ROS levels (Figure 1A). These findings indicate that *M. officinalis* extract was the sole component to exhibit antioxidant activity among the four extracts that are recognized as useful in skin care.

The finding that *M. officinalis* extract was effective in reducing ROS led to experiments to determine the optimal concentration at which *M. officinalis* extract reduced senescence–associated phenotypes. Since cell cycle arrest is one of the major hallmarks of senescence [17], the anti-senescence effect of *M. officinalis* extract was investigated based on whether *M. officinalis* extract has a cell proliferation-inducing effect. To find the optimal concentration for inducing cell proliferation effects, senescent fibroblasts were treated with *M. officinalis* at concentrations of 0.625, 1.25, 2.5, 5, and 10 μg/ml. Among the various concentrations, the cell proliferation induction effect was found at 0.625 and 1.25 μg/ml (Figure 1B). However, there was no effect at 2.5 μg/ml, and a decrease in cell proliferation was observed at 5 and 10 μg/ml (Figure 1B). These differences may be due to drug toxicity when the drug concentration exceeds the availability of intracellular drug receptors [29], or the failure of the drug to produce the desired pharmacological response when the drug concentration does not reach the availability of receptors [30]. Therefore, 0.625 μg/ml was selected as the optimal concentration of *M. officinalis* extract since it was the lowest concentration that causes cell proliferation.

Because the optimal concentration of *M. officinalis* extract (0.625 μg/ml) was different from the concentration (10 μg/ml) used in the ROS–based screening (Figure 1A), we investigated whether the ROS–lowering effect was maintained even at the optimal concentration. In addition to DHR123, MitoSox, which can detect O_2_^●−^ present in mitochondria, was used [31]. Senescent fibroblasts treated with DMSO had significantly higher mitochondrial ROS levels than young fibroblasts, whereas treatment with *M. officinalis* significantly reduced mitochondrial ROS levels in senescent fibroblasts (Figure 1C, 1D). These results suggest that the ROS–lowering effect of *M. officinalis* extract was reproducible even at 0.625 μg/ml.

Next, determining the optimal concentration of *M. officinalis* led us to investigate the toxicity of *M. officinalis* at the selected concentration. Cell proliferation measurement evaluates the rate at which cells divide in response to a drug [32], while cell viability measurement evaluates the percentage of living cells in response to a drug [33]. Therefore, cell viability was measured to assess whether *M. officinalis* extract was toxic to cells. The viability of *M. officinalis* extract-senescent fibroblasts was comparable to that of DMSO-treated senescent fibroblasts, indicating that *M. officinalis* at the selected concentration was not cell–toxic (Figure 1E).

### *M. officinalis* extract reduces mitochondrial ROS generation through mitochondrial functional recovery

**Figure 2:**
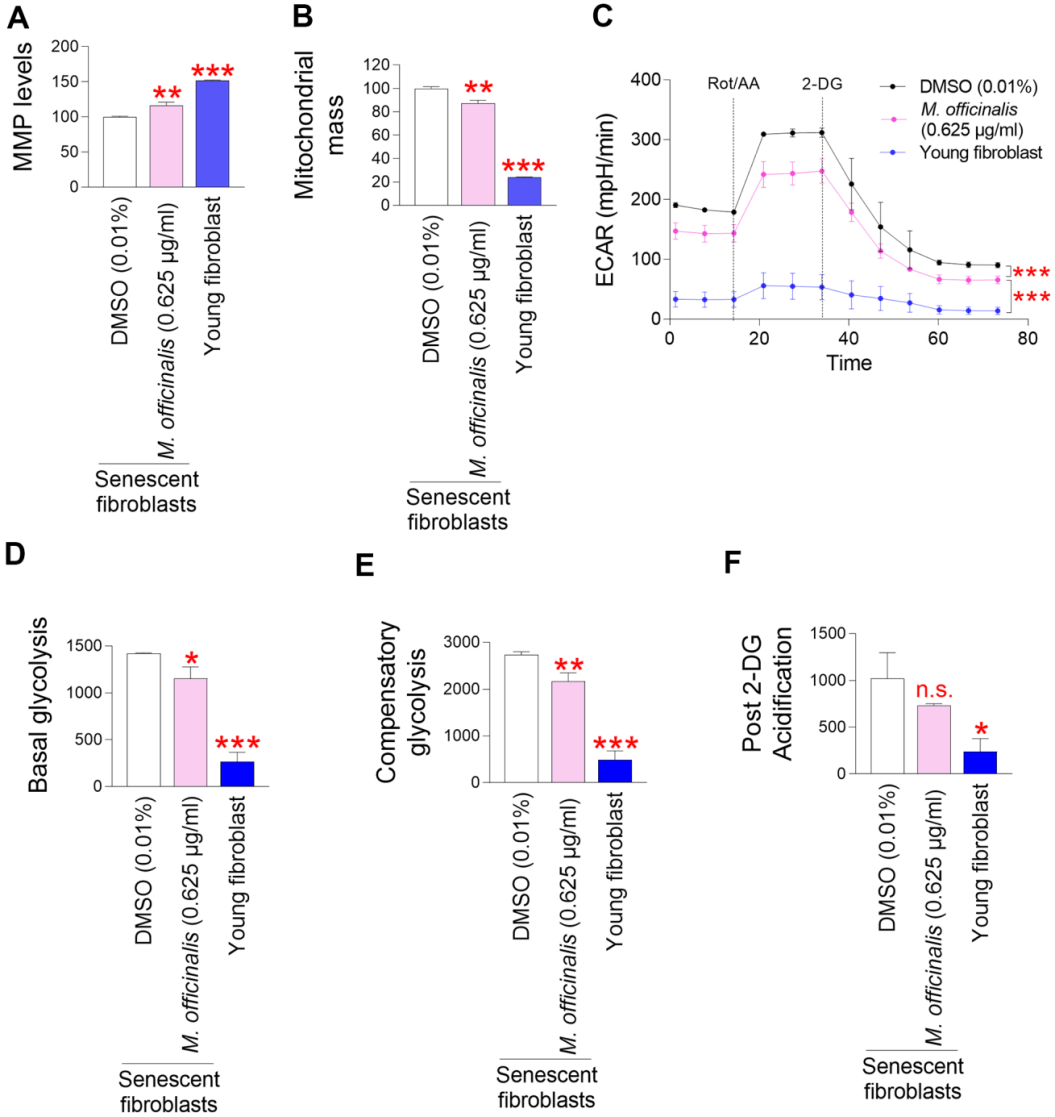
***M. officinalis* extract reduces mitochondrial ROS generation through mitochondrial functional recovery.** (**A**) Use of JC–10 for flow cytometric measurement of mitochondrial membrane potential (MMP). Senescent fibroblasts were treated with DMSO (0.01%) or *M. officinalis* extract (0.625 μg/ml) for 12 days. Young fibroblasts were used as a positive control. **P < 0.01, ***P < 0.001, Student's t–test. Mean ± S.D., N = 3. (**B**) MitoTracker green was employed for a flow cytometric study of mitochondrial mass. Senescent fibroblasts were treated with DMSO (0.01%) or *M. officinalis* extract (0.625 μg/ml) for 12 days. Young fibroblasts were used as a positive control. **P < 0.01, ***P < 0.001, Student t–test. Mean ± S.D., N = 3. (**C**) Measurement of extracellular acidification rate (ECAR; mpH/min) after 12 days of treatment with DMSO (0.01%) or *M. officinalis* extract (0.625 μg/ml) (black line: DMSO–treated senescent fibroblasts, pink line: *M. officinalis* extract–treated senescent fibroblasts). Young fibroblasts were used as a positive control. ***P < 0.001, two–way ANOVA followed by Bonferroni’s post–hoc test. Means ± S.D., N = 3. (**D**) Basal glycolysis was measured after 12 days of treatment with DMSO (0.01%) or *M. officinalis* extract (0.625 μg/ml). Young fibroblasts were used as a positive control. *P < 0.05, ***P < 0.001, Student t–test. Mean ± S.D., N = 3. (**E**) Compensatory glycolysis was measured after 12 days of treatment with DMSO (0.01%) or *M. officinalis* extract (0.625 μg/ml). Young fibroblasts were used as a positive control. **P < 0.01, ***P < 0.001, Student's t–test. Mean ± S.D., N = 3. (**F**) Post–2–DG acidification was measured after 12 days of treatment with DMSO (0.01%) or *M. officinalis* extract (0.625 μg/ml). Young fibroblasts were used as a positive control. n.s. (not significant), *P < 0.05, Student's t–test. Mean ± S.D., N = 3.

A major factor contributing to ROS generation in the mitochondrial ETC is inefficient electron transport [19]. Electron transport abnormalities cause ETC components to generate O_2_^●−^ [19]. ROS-induced damage to mitochondria reduces the mitochondrial membrane potential (MMP) produced by the movement of protons from the mitochondrial matrix to the intermembrane space [34]. Thus, inefficient electron transport increases mitochondrial ROS generation and decreases MMP, whereas efficient electron transport decreases mitochondrial ROS generation and increases MMP [35]. As we observed a decrease in ROS levels by *M. officinalis* extract, we investigated the effect of *M. officinalis* extract on MMP. Senescent fibroblasts treated with DMSO had significantly lower MMP than young fibroblasts, confirming the senescence-associated decrease in MMP [36] (Figure 2A). However, as compared to senescent fibroblasts treated with DMSO, those treated with *M. officinalis* extract exhibited a significant rise in MMP (Figure 2A).

Mitochondrial dysfunction results in a compensatory rise in mitochondrial volume [37]. Therefore, the increase in MMP by *M. officinalis* prompted us to measure mitochondrial mass. DMSO-treated senescent fibroblasts had significantly higher mitochondrial mass than young fibroblasts, consistent with previous findings [38, 39] (Figure 2B). However, senescent fibroblasts treated with *M. officinalis* extract showed a decrease in mitochondrial mass compared to the DMSO control, indicating restored mitochondrial function by *M. officinalis* extract (Figure 2B).

As mitochondrial function deteriorates in senescent cells, reliance on glycolysis as an energy source increases to meet energy requirements [40]. Thus, restoration of mitochondrial function reduces the dependence on glycolysis as a cellular energy source in senescent fibroblasts [41]. Therefore, the observation of restoration of mitochondrial function by *M. officinalis* prompted us to examine changes in glycolysis dependence. The extracellular acidification rate (ECAR) after sequential injections of rotenone/antimycin A (Rot/AA) and 2-deoxy-D-glucose (2-DG) was used to determine the glycolysis rate. Particularly, the basal glycolysis rate (before to Rot/AA injection), compensatory glycolysis (post-Rot/AA injection), and post-2-DG acidification (post-2-DG injection) were all estimated using the successively measured ECAR values. Senescent fibroblasts treated with DMSO had significantly higher ECAR values than young fibroblasts, consistent with senescence-associated increase in glycolysis rate [38, 39] (Figure 2C). However, senescent fibroblasts treated with *M. officinalis* extract had lower ECAR values than those treated with DMSO, suggesting that *M. officinalis* treatment lowed glycolysis rate (Figure 2C). Specifically, senescent fibroblasts treated with DMSO had significantly higher basal levels of glycolysis than young fibroblasts, whereas *M. officinalis* treatment significantly reduced basal levels of glycolysis, suggesting that the rate of conversion of glucose to lactate was reduced by treatment with *M. officinalis* [42] (Figure 2D). Addition of a mitochondrial inhibitor (Rot/AA), which inhibits oxidative phosphorylation and induces a compensatory shift toward using glycolysis, resulted in higher compensatory glycolysis in senescent fibroblasts treated with DMSO than young fibroblasts (Figure 2E). However, *M. officinalis* treatment significantly reduced compensatory glycolysis, suggesting that senescent fibroblasts treated with *M. officinalis* rely less on glycolysis to meet cellular energy demands (Figure 2E). Addition of 2–DG, which inhibits glycolysis, resulted in higher post 2–DG acidification in senescent fibroblasts treated with DMSO than young fibroblasts (Figure 2F). However, *M. officinalis* treatment significantly reduced post 2–DG acidification, indicating that *M. officinalis*-treated senescent fibroblasts have reduced residual glycolysis, which is not entirely suppressed by 2-DG [43] (Figure 2F). These results suggest that *M. officinalis* reduced the reliance on glycolysis as a cellular energy source, indicating restoration of mitochondrial function by *M. officinalis*.

In summary, our results indirectly imply the reduction in ROS levels by *M. officinalis* extract is a consequence of diminished mitochondrial ROS generation, which is facilitated by the restoration of mitochondrial function.

### Senescence–associated phenotypes are ameliorated by *M. officinalis* extract

**Figure 3:**
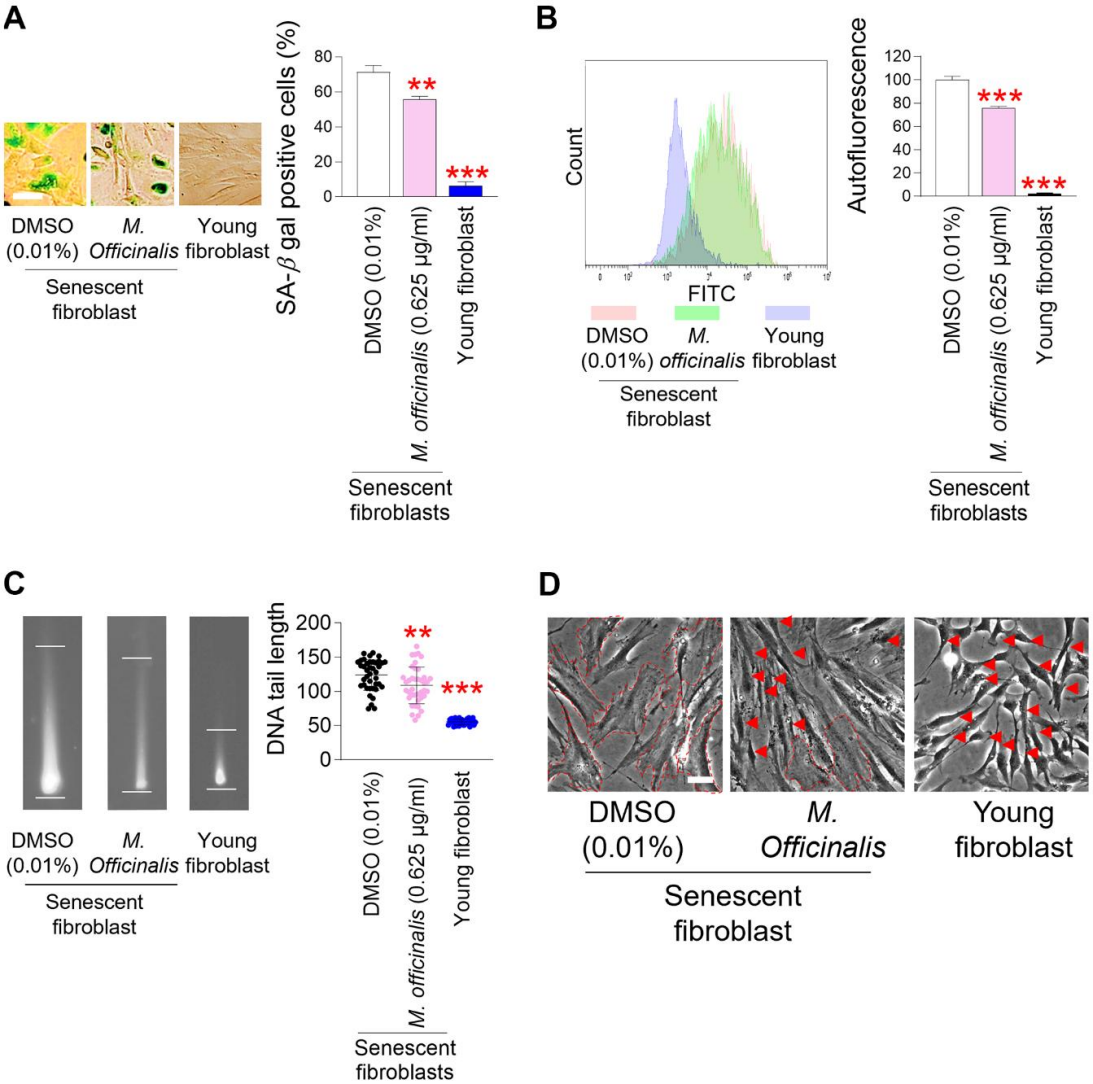
**Senescence–associated phenotypes are ameliorated by *M. officinalis* extract.** (**A**) Measurement of senescence–associated beta–galactosidase (SA–β–gal) positive cells (%). Senescent fibroblasts were treated with DMSO (0.01%) or *M. officinalis* extract (0.625 μg/ml) for 12 days. Young fibroblasts were used as a positive control. **P < 0.01, ***P < 0.001, Student’s t–test. Means ± S.D., N = 3. Scale bar 20 μm. (**B**) After 12 days of treatment with DMSO (0.01%) or *M. officinalis* extract (0.625 μg/ml), autofluorescence was assessed in senescent fibroblasts. Young fibroblasts were used as a positive control. **P < 0.01, ***P < 0.001, Student’s t–test. Means ± S.D., N = 3. (**C**) After 12 days of treatment with DMSO (0.01%) or *M. officinalis* extract (0.625 μg/ml), DNA tail length was assessed in senescent fibroblasts by image J. Each dot represents the length of a DNA tail. Young fibroblasts were used as a positive control. **P < 0.01, ***P < 0.001, Student's t–test. Mean ± S.D., N = 40. (**D**) Morphologies of senescence fibroblasts after 12 days of treatment with DMSO (0.01%) or *M. officinalis* extract (0.625 μg/ml). Senescent fibroblasts treated with *M. officinalis* extract exhibited thin and spindly morphology (red arrows), whereas those treated with DMSO exhibited a broad and flat morphology (dotted lines). The scale bar is 20 μm. Young fibroblasts were used as a positive control.

The restoration of mitochondrial function is one of the prerequisites for the amelioration of senescence [38, 40, 44–46]. The discovery of mitochondrial functional recovery by *M. officinalis* extract led to an investigation of the effect of *M. officinalis* on senescence-associated phenotypes. First, we investigated the effect of *M. officinalis* on senescence–associated beta–galactosidase (SA–β–gal), a characteristic trait of senescence [47]. DMSO-treated senescent fibroblasts had significantly higher percentage of SA–β–gal positive cells than young fibroblasts, whereas *M. officinalis* treatment significantly reduced the percentage of SA–β–gal positive cells (Figure 3A).

Then, the amount of accumulated intracellular lipofuscin, a characteristic of senescence–associated phenotypes, was evaluated by measuring intracellular autofluorescence levels [48]. Senescent fibroblasts treated with DMSO had significantly higher autofluorescence levels than young fibroblasts, consistent with senescence-associated increase in autofluorescence levels [39] (Figure 3B). However, the autofluorescence level was significantly reduced after *M. officinalis* treatment, suggesting that *M. officinalis* reduced intracellular lipofuscin levels (Figure 3B).

ROS cause DNA damage, which is known as a mediator of senescence, either directly affecting DNA or affecting proteins involved in DNA maintenance [49]. Since we observed *M. officinalis* extract–mediated ROS reduction, the effect of *M. officinalis* extract on DNA damage was evaluated. To identify the presence of DNA damage, DNA double–strand breaks (DSBs) was examined [50]. As expected, senescent fibroblasts treated with DMSO showed higher DNA DSBs than young fibroblasts [39, 51], whereas *M. officinalis* treated-senescent fibroblasts significantly diminished DNA DSBs in comparison to the DMSO control (Figure 3C).

An increase in cell surface area is a major characteristic associated with senescence [4]. Thus, morphological changes in senescent fibroblasts were analyzed after treatment with *M. officinalis*. DMSO–treated senescent fibroblasts exhibited a broad and flat morphology (dotted lines), while *M. officinalis*–treated senescent fibroblasts displayed a thin and spindly morphology (red arrows), a characteristic typically observed in young fibroblasts (Figure 3D). To objectively quantify the results in Figure 3D, we evaluated the forward scatter (FSC) levels of cells. Since FSC levels are proportional to cell diameter, they have been widely used to examine cell size [52]. Senescent fibroblasts treated with DMSO had significantly higher FSC levels than young fibroblasts, supporting the senescence-associated increase in cell surface area [4] (Supplementary Figure 1). In addition, senescent fibroblasts treated with *M. officinalis* had significantly decreased FSC levels compared to DMSO-treated senescent fibroblasts, confirming the results in Figure 3E that *M. officinalis* increased the proportion of thin and slender cell morphologies (Supplementary Figure 1).

### *M. officinalis* extract enhances skin barrier function by increasing skin regeneration and suppressing skin inflammation

The skin is composed of the epidermis, the outer layer that mainly contains keratinocytes, and the dermis, the underlying layer that mainly contains connective tissue [53]. The skin is one of the areas where aging is most noticeable [54]. The finding that *M. officinalis* extract improves senescence–associated phenotypes prompted us to investigate whether *M. officinalis* extract might have any effect on skin protection.

**Figure 4:**
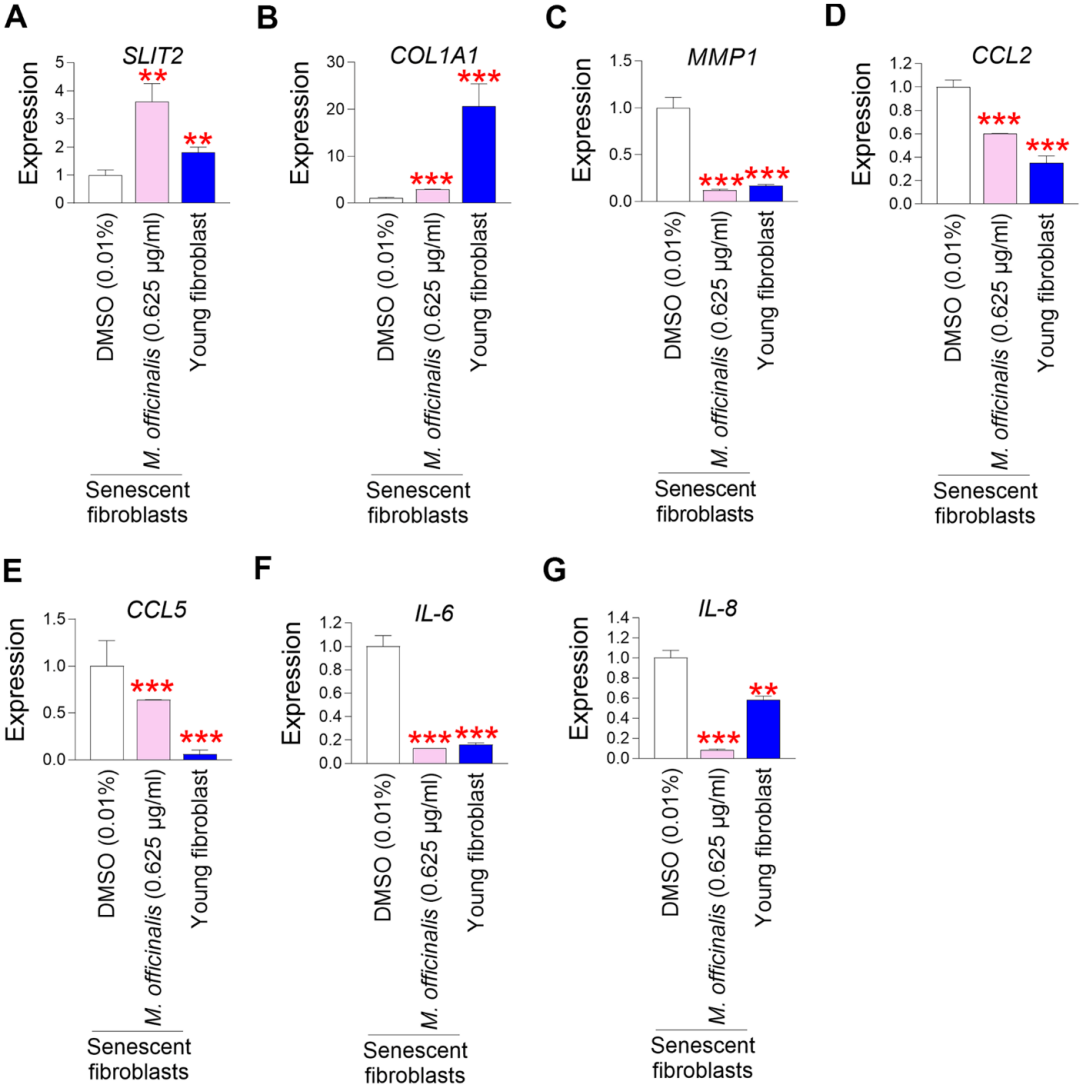
***M. officinalis* extract enhances skin barrier function by increasing skin regeneration and suppressing skin inflammation.** (**A**) The expression levels of the *SLIT2* in senescent fibroblasts were assessed after 12 days of treatment with DMSO (0.01%) or *M. officinalis* extract (0.625 μg/ml). Young fibroblasts were used as a positive control. **P < 0.01, Student’s t–test. Means ± S.D., N = 3. (**B**) The expression levels of the *COL1A1* in senescent fibroblasts were assessed after 12 days of treatment with DMSO (0.01%) or *M. officinalis* extract (0.625 μg/ml). Young fibroblasts were used as a positive control. ***P < 0.001, Student’s t–test. Means ± S.D., N = 3. (**C**) The expression levels of the *MMP1* in senescent fibroblasts were assessed after 12 days of treatment with DMSO (0.01%) or *M. officinalis* extract (0.625 μg/ml). Young fibroblasts were used as a positive control. ***P < 0.001, Student’s t–test. Means ± S.D., N = 3. (**D**) The expression levels of the *CCL2* in senescent fibroblasts were assessed after 12 days of treatment with DMSO (0.01%) or *M. officinalis* extract (0.625 μg/ml). Young fibroblasts were used as a positive control. ***P < 0.001, Student’s t–test. Means ± S.D., N = 3. (**E**) The expression levels of the *CCL5* in senescent fibroblasts were assessed after 12 days of treatment with DMSO (0.01%) or *M. officinalis* extract (0.625 μg/ml). Young fibroblasts were used as a positive control. ***P < 0.001, Student’s t–test. Means ± S.D., N = 3. (**F**) The expression levels of the *IL–6* in senescent fibroblasts were assessed after 12 days of treatment with DMSO (0.01%) or *M. officinalis* extract (0.625 μg/ml). Young fibroblasts were used as a positive control. ***P < 0.001, Student’s t–test. Means ± S.D., N = 3. (**G**) The expression levels of the *IL–8* in senescent fibroblasts were assessed after 12 days of treatment with DMSO (0.01%) or *M. officinalis* extract (0.625 μg/ml). **P < 0.01, Student’s t–test. Mean ± S.D., N = 3. Young fibroblasts were used as a positive control. **P < 0.01, ***P < 0.001, Student’s t–test. Means ± S.D., N = 3.

*Slit guidance ligand 2* (*SLIT2*) contributes to restoring skin tissue regeneration by regulating cell–cell interactions [55, 56]. Downregulation of *SLIT2* expression impairs skin barrier function by reducing tissue regenerative capacity [55, 57]. To examine the role of *M. officinalis* extract on *SLIT2* expression, *M. officinalis* extract was treated to senescent fibroblasts. Senescent fibroblasts treated with DMSO had significantly lower *SLIT2* expression than young fibroblasts, but treatment with *M. officinalis* significantly increased *SLIT2* expression in senescent fibroblasts, indicating that *M. officinalis* promotes skin regeneration (Figure 4A).

A distinguishing feature of skin aging is a decrease in collagen protein synthesis, which causes the skin to lose structural support and its barrier function to decline [58]. To determine the effect of *M. officinalis* extract on collagen production, the expression level *of type I collagen alpha 1* (*COL1A1*) was analyzed. Senescent fibroblasts treated with DMSO had significantly lower *COL1A1* expression than young fibroblasts, but treatment with *M. officinalis* significantly increased *COL1A1* expression in senescent fibroblasts, indicating that *M. officinalis* enhances structural support (Figure 4B).

Skin barrier function is deteriorated by an increase in matrix *metalloprotease 1* (*MMP1*) expression, which promotes collagen degradation [59]. The discovery that *M. officinalis* extract enhanced collagen production led us to examine its impact on *MMP1* expression. Senescent fibroblasts treated with DMSO had significantly higher *MMP1* expression than young fibroblasts, but treatment with *M. officinalis* significantly decreased *MMP1* expression in senescent fibroblasts, indicating that *M. officinalis* prevents collagen degradation (Figure 4C).

Skin inflammation is a primary contributor to compromised skin barrier function, as it facilitates collagen degradation and diminishes regenerative capacity [60]. C*–C motif chemokine ligands* (*CCL*) induce skin inflammation by recruiting various leukocytes to the skin [61, 62]. The impact of *M. officinalis* extract on *CCL* expression was assessed by examining the expression of *CCL2* and *CCL5*. Senescent fibroblasts treated with DMSO had significantly higher expression of *CCL2* and *CCL5* than young fibroblasts, but treatment with *M. officinalis* significantly reduced the expression of *CCL2* and *CCL5* in senescent fibroblasts, indicating that *M. officinalis* extract reduces skin inflammation (Figure 4D, 4E).

The skin, which acts as an immune organ, activates the activity of cytokines, including interleukin, in response to attacks by various pathogens. *Interleukin–6* (*IL–6*) is synthesized in reaction to the infiltration of inflammatory cells in many skin areas, including fibroblasts, epidermal cells, and dermal endothelial cells [63]. *IL–8* is an inflammatory mediator that significantly contributes to skin inflammation [64]. To determine the role of *M. officinalis* extract on interleukin expression, the expression of *IL-6* and *IL-8* was investigated. Senescent fibroblasts treated with DMSO had significantly higher expression of *IL-6* and *IL-8* than young fibroblasts (Figure 4F, 4G). However, treatment with *M. officinalis* significantly decreased the expression of *IL-6* and *IL-8* in senescent fibroblasts, suggesting that *M. officinalis* extract lowers the expression of inflammatory cytokines (Figure 4F, 4G).

### *M. officinalis* extract reverses skin aging by suppressing skin pigmentation and maintaining skin moisture

**Figure 5:**
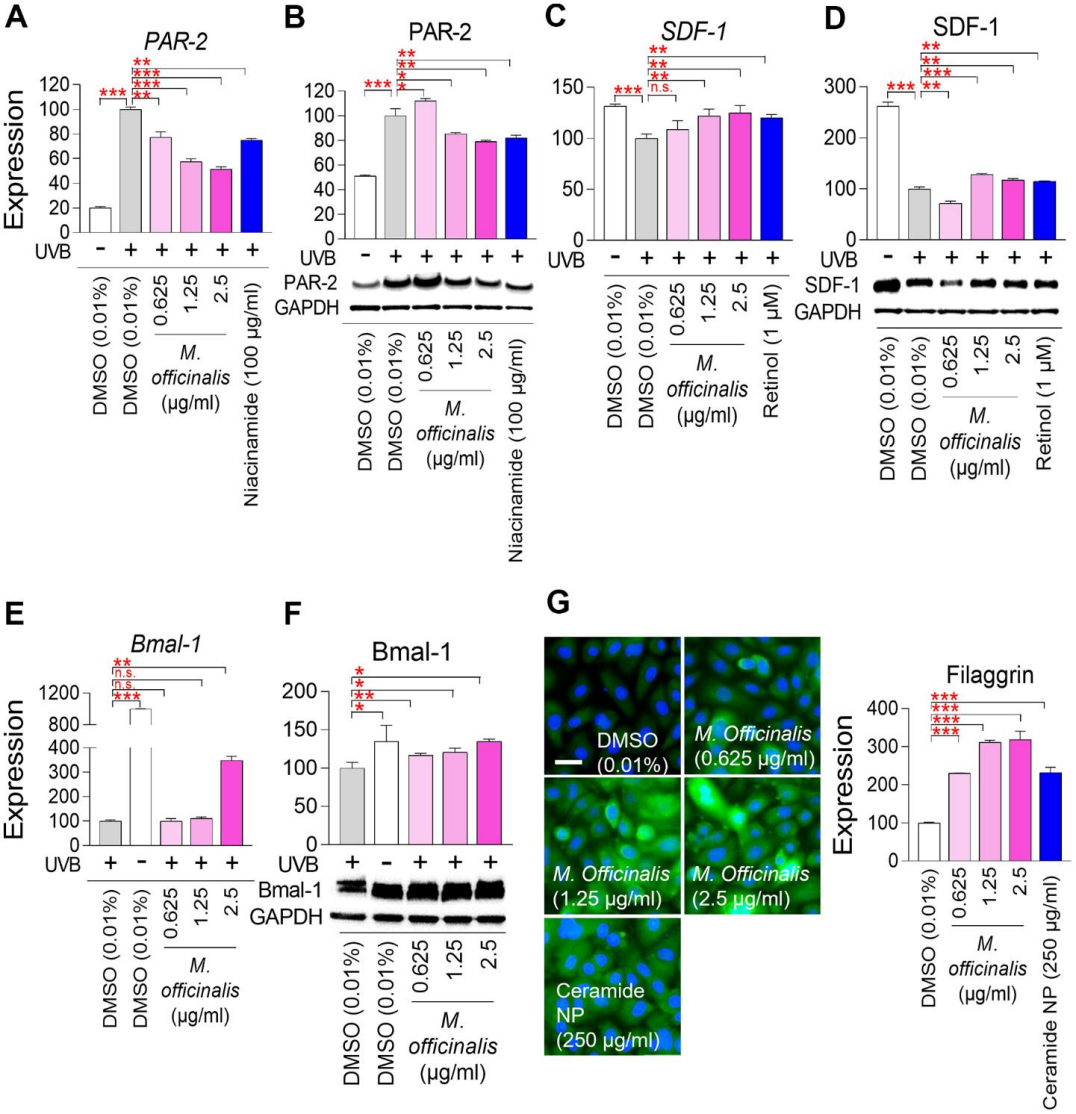
***M. officinalis* extract reverses skin aging by suppressing skin pigmentation, increasing skin turnover, and maintaining skin moisture.** (**A**, **B**) Measurement of melanosome transport. To stimulate melanosome production, HaCaT cells were exposed to 30 mJ/cm^2^ ultraviolet B (UVB). Then, HaCaT cells were treated with DMSO (0.01%) or *M. officinalis* extract (0.625, 1.25, and 2.5 μg/ml) for 8 h. As a positive control, 100 μg/ml niacinamide was used. PAR–2 expression was analyzed by qPCR (**A**) and Western blot (**B**). *P < 0.05, **P < 0.01, ***P < 0.001, Student’s t–test. Mean ± S.D., N = 3. (**C**, **D**) Measurement of skin pigmentation. To induce skin pigmentation, HaCaT cells were exposed to 15 J/cm^2^ UVB. Then, HaCaT cells were treated with DMSO (0.01%) or *M. officinalis* extract (0.625, 1.25, and 2.5 μg/ml) for 24 h. As a positive control, 1 μM retinol (R7632; Sigma) was used. SDF–1 expression was analyzed by qPCR (**C**) and Western blot (**D**). n.s. (not significant), **P < 0.01, ***P < 0.001, Student’s t–test. Mean ± S.D., N = 3. (**E**, **F**) Measurement of skin turnover. To disrupt skin turnover, HaCaT cells were exposed to 30 mJ/cm^2^ UVB. Then, HaCaT cells were treated with DMSO (0.01%) or *M. officinalis* extract (0.625, 1.25, and 2.5 μg/ml) for 24 h. Bmal–1expression was analyzed by qPCR (**E**) and Western blot (**F**). n.s. (not significant), *P < 0.05, **P < 0.01, ***P < 0.001, Student’s t–test. Mean ± S.D., N = 3. (**G**) Measurement of skin moisture retention. Normal human epidermal keratinocytes, HEKn cells, were treated with DMSO (0.01%) or *M. officinalis* extract (0.625, 1.25, and 2.5 μg/ml) for 72 h. As a positive control, 250 μg/ml ceramide NP was used. Expression of filaggrin protein was examined using immunocytochemistry (blue: dapi, green: filaggrin). Image J analysis was performed to quantify the fluorescence intensity of filaggrin protein. ***P < 0.001, Student’s t–test. Mean ± S.D., N = 3. The scale bar is 100 μm.

Skin pigmentation is a hallmark of aging and results from the accumulation of melanosomes, organelles that contain melanin, the pigment responsible for skin color, within keratinocytes [65]. *Protease–activated receptor–2* (*PAR–2*) has an important function in skin pigmentation by transporting melanosomes from melanocytes to keratinocytes [66]. Overactivation of *PAR–2* can lead to increased melanosome transport, which can cause hyperpigmentation [66]. To examine the effect of *M. officinalis* extract on skin pigmentation, the mRNA and protein expression of PAR*–*2, were evaluated. HaCaT cells were irradiated with ultraviolet B (UVB), which stimulates melanosome production [67]. Niacinamide, recognized for its ability to reduce PAR–2 expression, served as a positive control [68]. Irradiation of HaCaT cells by UVB significantly upregulated the mRNA and protein expression of PAR*–*2 (Figure 5A, 5B). However, niacinamide treatment significantly decreased the mRNA and protein expression of PAR*–*2 (Figure 5A, 5B). Furthermore, the mRNA expression levels of *PAR–2* were significantly downregulated by *M. officinalis* at 0.625, 1.25, and 2.5 μg/ml, and the protein expression levels of PAR*–*2 were significantly downregulated by *M. officinalis* at 1.25 and 2.5 μg/ml (Figure 5A, Figure 15B). These results suggest that downregulation of PAR–2 expression by *M. officinalis* may reduce skin pigmentation.

*Stromal cell–derived factor 1* (*SDF–1*) reduces skin pigmentation by inhibiting the cAMP signaling pathway in melanocytes [69]. To further investigate the role of *M. officinalis* extract on skin pigmentation, the mRNA and protein expression of SDF–1, were analyzed after UVB irradiation of HaCaT cells. Retinol, recognized for its ability to restore SDF–1 expression, served as a positive control [68]. Irradiation of HaCaT cells by UVB significantly increased the mRNA and protein expression of SDF–1 (Figure 5C, Figure 5D). However, retinol treatment significantly decreased the mRNA and protein expression of SDF–1 (Figure 5C, Figure 5D). Furthermore, the mRNA and protein expression levels of SDF–1 were significantly decreased by *M. officinalis* at 1.25 and 2.5 μg/ml (Figure 5C, Figure 5D). These results indicate that upregulation of SDF–1 expression by *M. officinalis* may reduce skin pigmentation.

*Brain and muscle ARNT–like 1* (*Bmal–1*) are essential for sustaining youthful and healthy skin by regulating cell proliferation and differentiation, therefore enhancing skin turnover [70]. To examine the role of *M. officinalis* extract on skin turnover, the mRNA and protein expression of Bmal–1, were analyzed after UVB irradiation of HaCaT cells. Irradiation of HaCaT cells by UVB significantly decreased the mRNA and protein expression of Bmal–1 (Figure 5E, Figure 5F). However, the mRNA and protein expression levels of Bmal–1 were significantly increased by *M. officinalis* at 2.5 μg/ml (Figure 5E, Figure 5F). These results suggest that the activation of Bmal–1 expression by *M. officinalis* promote skin turnover.

Filaggrin is a protein synthesized in keratinocytes and serves as a key component of the outermost layer of the skin, playing an essential function in maintaining skin hydration [71]. Skin hydration indicates the water content of the stratum corneum of the epidermis, and can be indirectly measured by measuring filaggrin expression [72]. HEKn cells are human epidermal keratinocytes and are closer to the stratum corneum than HaCaT cells, which are keratinocytes [73]. Consequently, the study of skin hydration has made extensive use of HEKn cells [74, 75]. Therefore, to examine the function of *M. officinalis* extract on skin hydration, HEKn cells were treated with *M. officinalis* extract and the changes in filaggrin protein expression were examined using immunocytochemistry. Ceramide–NP, which is well known to increase filaggrin expression, was used as a positive control [76]. As anticipated, ceramide NP significantly increased the level of filaggrin expression (Figure 5G). In HEKn cells treated with *M. officinalis*, a significant increase in filaggrin expression was observed at all concentrations (0.625, 1.25, and 2.5 μg/ml) compared to the DMSO control. These results suggest that the activation of filaggrin expression by *M. officinalis* promote skin hydration.

### Identification of honokiol as a core ingredient showing antioxidant effects

**Figure 6:**
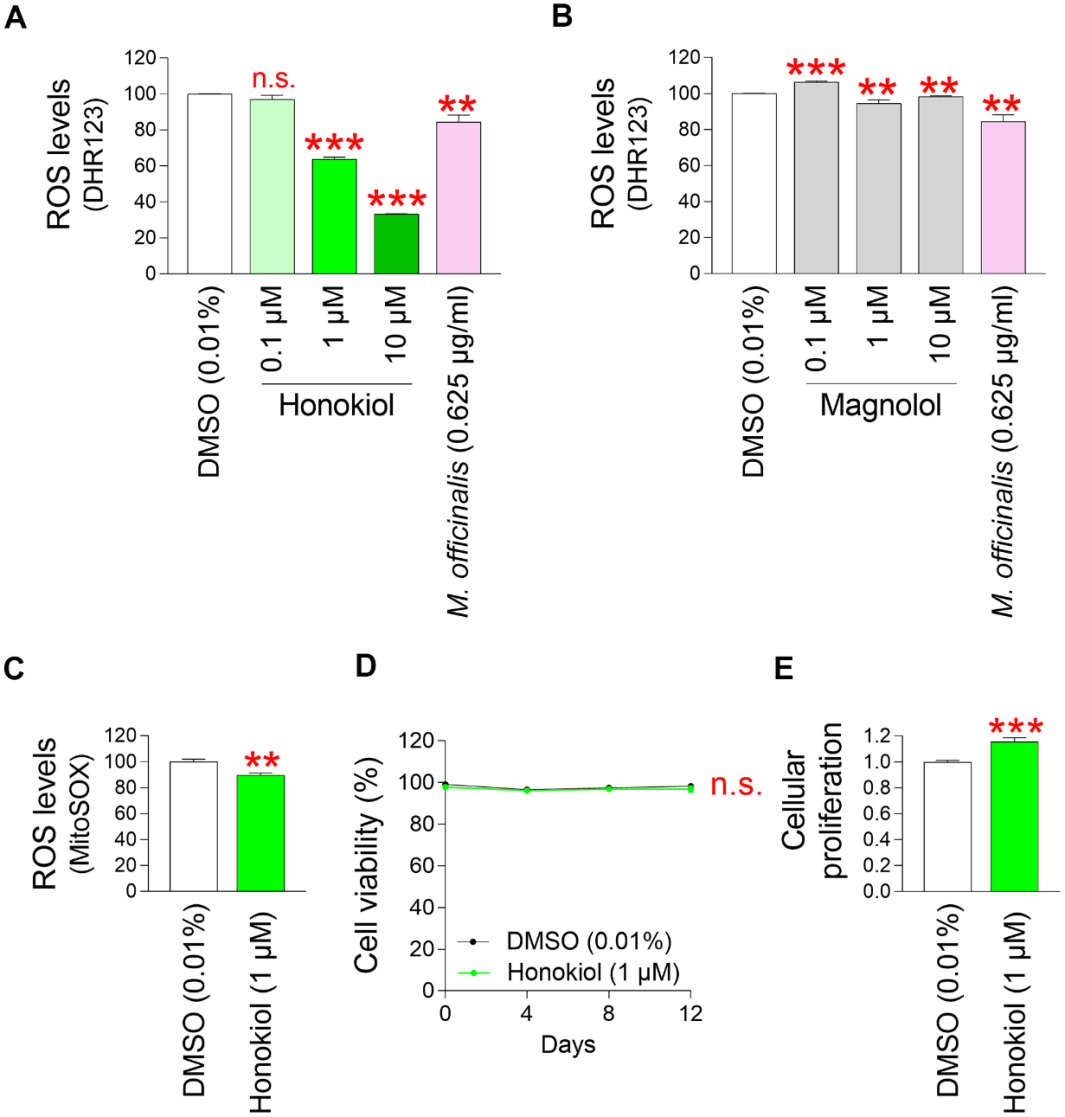
**Identification of honokiol as a core ingredient showing antioxidant effects.** (**A**) Senescent fibroblasts were treated with DMSO (0.01%) or honokiol (0.1, 1, and 10 μM) for 12 days. Use of DHR123 for flow cytometric analysis of mitochondrial ROS levels. The extract of *M. officinalis* extract (0.625 μg/ml) served as a positive control. Honokiol at 1 and 10 μM was more effective in reducing ROS levels than *M. officinalis* extract. n.s. (not significant), **P < 0.01, ***P < 0.001, Student’s t–test. Mean ± S.D., N = 3. (**B**) Senescent fibroblasts were treated with DMSO (0.01%) or magnolol (0.1, 1, and 10 μM) for 12 days. Use of DHR123 for flow cytometric analysis of mitochondrial ROS levels. The extract of *M. officinalis* extract (0.625 μg/ml) served as a positive control. Magnolol at 1 and 10 μM was not more effective in reducing ROS levels than *M. officinalis* extract. n.s. (not significant), ***P < 0.001, **P < 0.01, Student’s t–test. Mean ± S.D., N = 3. (**C**) Senescent fibroblasts were treated with DMSO (0.01%) or honokiol (1 μM). Measurement of cell viability after 0, 4, 8, and 12 days of treatment. n.s. (not significant), two–way ANOVA followed by Bonferroni’s post–hoc test. Mean ± S.D., N = 3. (**D**) Cellular proliferation was evaluated at DMSO (0.01%) or honokiol (1 μM) on day 12 after treatment in senescent fibroblasts. ***P < 0.001, Student’s t–test. Mean ± S.D., N = 3. (**E**) Senescent fibroblasts were treated with DMSO (0.01%) or honokiol (1 μM) for 12 days. Use of MitoSOX for flow cytometric analysis of mitochondrial ROS levels. **P < 0.01, Student's t–test. Mean ± S.D., N = 3.

After discovering that *M. officinalis* extract is effective in protecting skin and reversing skin aging, we decided to investigate which components present in *M. officinalis* extract are responsible for these effects. Previous studies have found that the major components of *M. officinalis* are honokiol and magnolol [77]. Honokiol, classified as a lignan, contains natural phenolic cytotoxic compounds that serve as oxygen radical scavengers through redox reactions [78]. Magnolol, classified as a lignan, has antioxidant and antibacterial effects by interfering with NF–κB signaling [79]. To identify core ingredients that have antioxidant properties, senescent fibroblasts were subjected to treatment with varying concentrations of honokiol and magnolol. Subsequently, their impact on ROS levels was assessed using DHR123. The extract of *M. officinalis* extract served as a positive control. Not significantly reducing mitochondrial ROS levels was observed at a 0.1 μM honokiol, whereas significantly reducing ROS levels were observed at 1 and 10 μM (Figure 6A). Moreover, compared to *M. officinalis* extract, honokiol at 1 and 10 μM was more effective in lowering mitochondrial ROS levels (Figure 6A). However, magnolol at 0.1 μM significantly increased mitochondrial ROS levels compared to DMSO control (Figure 6B). Significant reducing mitochondrial ROS levels were observed at 1 and 10 μM magnolol, whereas magnolol at those concentrations was not more effective in lowering mitochondrial ROS levels than *M. officinalis* extract (Figure 6B). Based on these data, honokiol is the key substance exhibiting antioxidant properties among various components of *M. officinalis* extract. The least honokiol concentration that was more efficient than *M. officinalis* extract was chosen as the optimal concentration for the further studies, which was 1 μM.

Then, the ROS-reducing effect of honokiol was re-examined using MitoSox. Senescent fibroblasts treated with honokiol showed significantly lowered mitochondrial ROS levels compared to senescent fibroblasts treated with DMSO (Figure 6C).

Next, we investigated the toxicity of 1 μM honokiol by investigating cell viability. The cell viability of senescent fibroblasts treated with 1 μM honokiol was comparable to that of senescent fibroblasts treated with DMSO, suggesting that honokiol was non–toxic to the cells at selected concentration (Figure 6D).

Finally, we performed a cell proliferation assay to confirm the senescence ameliorating effect of honokiol. Senescent fibroblasts treated with honokiol exhibited a cell proliferation-induction effect similar to that of *M. officinalis* extract (Figure 6E).

### Honokiol reduces mitochondrial ROS generation through mitochondrial functional recovery

We found that *M. officinalis* extract reduced mitochondrial ROS production by restoring mitochondrial function. Therefore, we investigated whether honokiol, the main component of this extract, also had the same effect.

**Figure 7:**
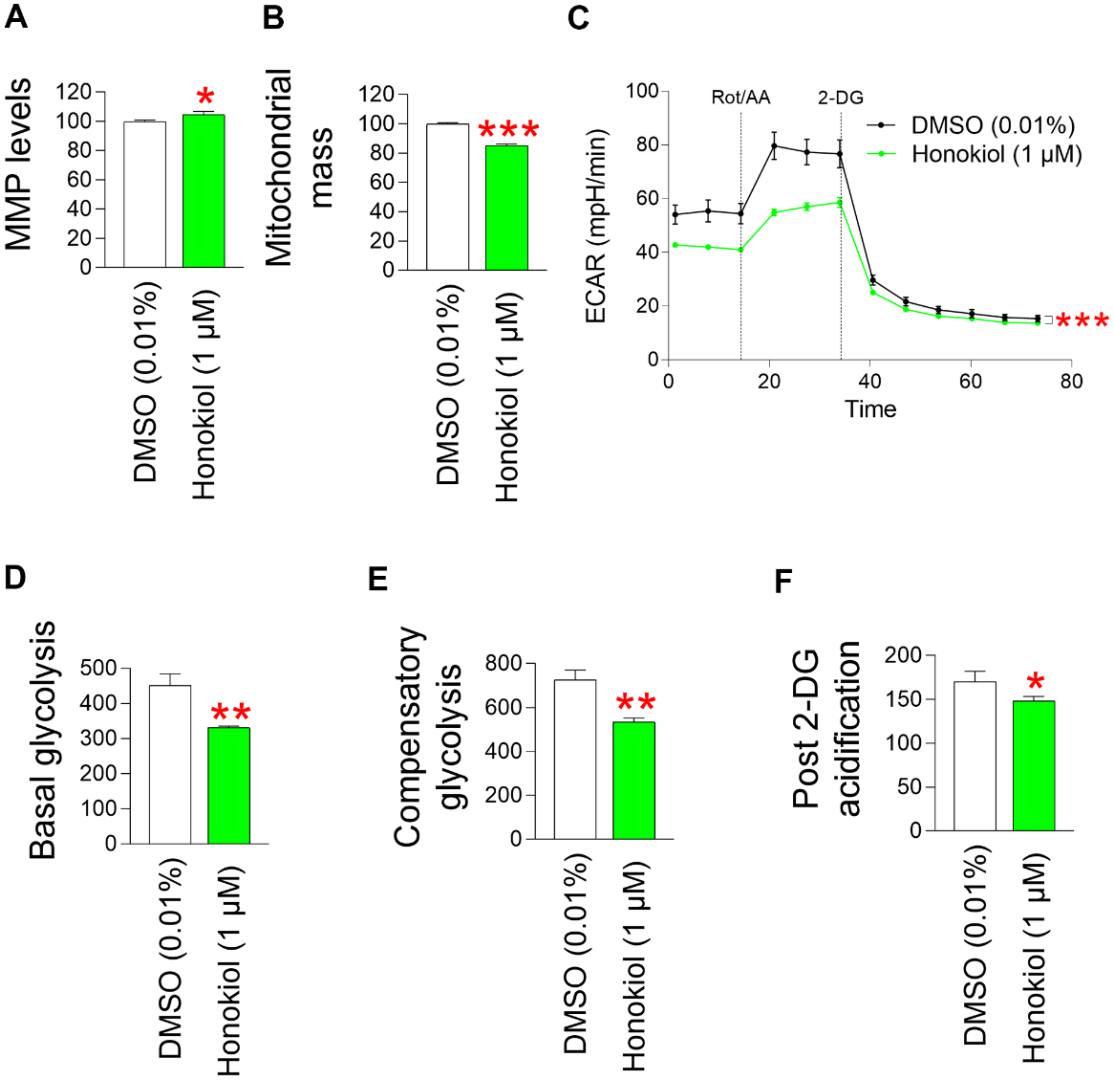
**Honokiol reduces mitochondrial ROS generation through mitochondrial functional recovery.** (**A**) Use of JC–10 for flow cytometric measurement of MMP. Senescent fibroblasts were treated with DMSO (0.01%) or honokiol (1 μM) for 12 days. *P < 0.05, Student's t–test. Mean ± S.D., N = 3. (**B**) MitoTracker green was employed for a flow cytometric study of mitochondrial mass. Senescent fibroblasts were treated with DMSO (0.01%) or honokiol (1 μM) for 12 days. ***P < 0.001, Student t–test. Mean ± S.D., N = 3. (**C**) Measurement of extracellular acidification rate (ECAR; mpH/min) after 12 days of treatment with DMSO (0.01%) or 1 μM honokiol. (black line: DMSO–treated senescent fibroblasts, pink line: honokiol–treated senescent fibroblasts). ***P < 0.001, two–way ANOVA followed by Bonferroni’s post–hoc test. Means ± S.D., N = 3. (**D**) Basal glycolysis was measured after 12 days of treatment with DMSO (0.01%) or honokiol (1 μM). **P < 0.01, student t–test. Mean ± S.D., N = 3. (**E**) The compensatory glycolysis was measured after 12 days of treatment with DMSO (0.01%) or honokiol (1 μM). **P < 0.01, Student t–test. Mean ± S.D., N = 3. (**F**) Post–2–DG acidification was measured after 12 days of treatment with DMSO (0.01%) or honokiol (1 μM). *P < 0.05, Student t–test. Mean ± S.D., N = 3.

The finding that honokiol reduces mitochondrial ROS levels in senescent fibroblasts conjectured us to investigate the effects of honokiol on MMP. Senescent fibroblasts treated with honokiol exhibited a significant rise in MMP relative to those treated with DMSO (Figure 7A). The rise in MMP by honokiol led to a measurement of mitochondrial mass. Honokiol-treated senescent fibroblasts exhibited a decrease in mitochondrial mass relative to the DMSO control (Figure 7B). These results support honokiol–mediated restoration of mitochondrial function.

Since *M. officinalis* extract reduced dependence on glycolysis as a cellular energy source, we investigated whether honokiol also reduced the dependence. Senescent fibroblasts treated with honokiol had lower ECAR values than DMSO–treated fibroblasts, suggesting a lower rate of glycolysis in honokiol–treated fibroblasts (Figure 7C). Specifically, senescent fibroblasts treated with honokiol had lower basal levels of glycolysis than DMSO controls, suggesting that honokiol reduced the rate at which glucose was converted to lactate (Figure 7D). Inhibition of oxidative phosphorylation and induction of a compensatory shift toward glycolysis resulted in lower compensatory glycolysis in honokiol–treated senescent fibroblasts compared to the DMSO control, suggesting that honokiol–treated senescent fibroblasts rely less on glycolysis to meet their energy requirements (Figure 7E). Addition of 2–DG reduced the post 2–DG acidification in honokiol–treated senescent fibroblasts compared to the DMSO control, suggesting that honokiol–treated senescent fibroblasts have lower residual glycolysis that is not completely blocked by 2–DG (Figure 7F). These results suggest that honokiol, similar to *M. officinalis*, reduced the dependence of glycolysis as an energy source, indicating restoration of mitochondrial function by honokiol.

In summary, our findings suggest that a reduction in ROS levels attributed to *M. officinalis* extract is a consequence of diminished mitochondrial ROS production, facilitated by honokiol–mediated mitochondrial functional recovery.

### Honokiol ameliorates senescence–associated phenotypes and enhances skin barrier function

**Figure 8:**
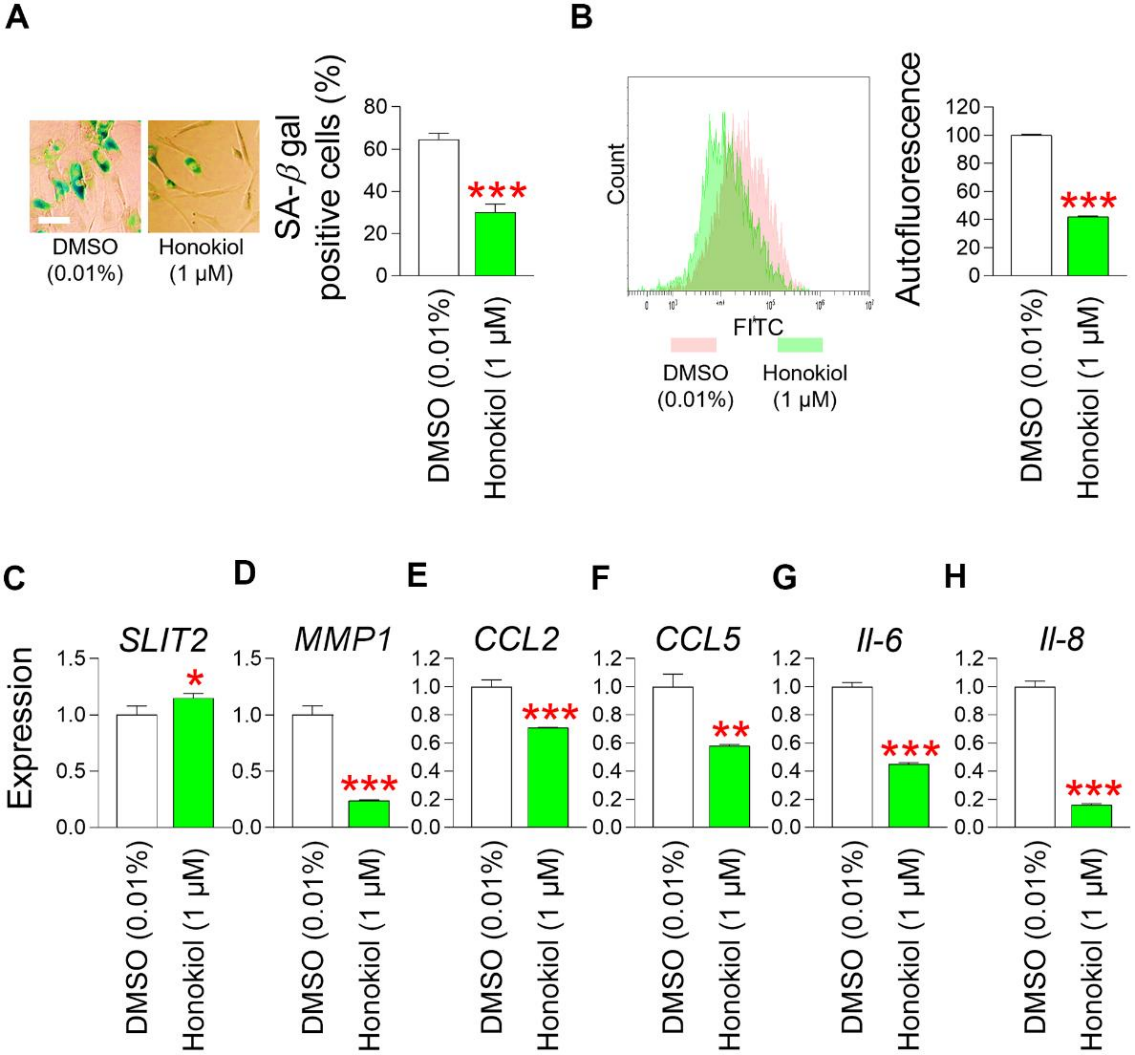
**Honokiol ameliorates senescence–associated phenotypes and enhances skin barrier function.** (**A**) Measurement of SA–β–gal positive cells (%). Senescent fibroblasts were treated with DMSO (0.01%) or honokiol (1 μM) for 12 days. ***P < 0.001, Student t–test. Mean ± S.D., N = 3. Scale bar 20 μm. (**B**) After 12 days of treatment with DMSO (0.01%) or honokiol (1 μM), autofluorescence was assessed in senescent fibroblasts by flow cytometry. The representative histogram of autofluorescence was shown. ***P < 0.001, Student t–test. Mean ± S.D., N = 3. (**C**) The expression levels of the *SLIT2* in senescent fibroblasts were assessed after 12 days of treatment with DMSO (0.01%) or honokiol (1 μM). *P < 0.05, Student t–test. Mean ± S.D., N = 3. (**D**) The expression levels of the *MMP1* in senescent fibroblasts were assessed after 12 days of treatment with DMSO (0.01%) or honokiol (1 μM). ***P < 0.001, Student t–test. Mean ± S.D., N = 3. (**E**) The expression levels of the *CCL2* in senescent fibroblasts were assessed after 12 days of treatment with DMSO (0.01%) or honokiol (1 μM). ***P < 0.001, Student t–test. Mean ± S.D., N = 3. (**F**) The expression levels of the *CCL5* in senescent fibroblasts were assessed after 12 days of treatment with DMSO (0.01%) or honokiol (1 μM). **P < 0.01, Student t–test. Mean ± S.D., N = 3. (**G**) The expression levels of the *IL–6* in senescent fibroblasts were assessed after 12 days of treatment with DMSO (0.01%) or honokiol (1 μM). ***P < 0.001, Student t–test. Mean ± S.D., N = 3. (**H**) The expression levels of the *IL–8* in senescent fibroblasts were assessed after 12 days of treatment with DMSO (0.01%) or honokiol (1 μM). ***P < 0.01, Student t–test. Mean ± S.D., N = 3.

The confirmation of mitochondrial functional recovery by honokiol prompted us to investigate the effects of honokiol on senescence–associated phenotypes. Treatment of senescent fibroblasts with honokiol significantly reduced the SA–β–gal positive cells (%) in comparison to the DMSO control (Figure 8A). In addition, autofluorescence was significantly reduced after honokiol treatment, suggesting that honokiol reduced intracellular lipofuscin levels (Figure 8B).

These data indicate that honokiol improves senescence–associated phenotypes.

The discovery that honokiol enhances senescence–associated phenotypes prompted us to explore whether honokiol, similar to *M. officinalis* extract, could contribute to the restoration of skin barrier function. Initially, we examined the influence of honokiol on *SLIT2* expression, which is crucial for restoring skin barrier function through the enhancement of skin regeneration. Honokiol significantly increased *SLIT2* expression compared to the DMSO control, indicating honokiol–mediated enhancement of skin regeneration (Figure 8C). We then investigated the effect of honokiol on *MMP1*, which impairs skin barrier function. Honokiol significantly decreased *MMP1* expression, indicating that honokiol maintains skin barrier function by inhibiting the expression of *MMP1*, which promotes collagen degradation (Figure 8D). Ultimately, we examined the impact of honokiol on skin inflammation, an important contributor to compromised skin barrier function. In senescent fibroblasts treated with honokiol, the expression of *CCL2* and *CCL5* was significantly decreased comparison to the DMSO control, indicating that honokiol reduced skin inflammation (Figure 8E, Figure 8F). Moreover, the expression levels of *IL–6* and *IL–8* were significantly lowered by honokiol in comparison to the DMSO control, suggesting that honokiol mitigates skin inflammation by reducing the expression of cytokines associated with inflammation (Figure 8G, Figure 8H).

## DISCUSSION

Damage to organelles induced by oxidative stress is closely related to the progression of senescence [80]. The major organelle generating ROS in cells is mitochondria [81]. The ETC complex in mitochondria converts 1%-5% of the oxygen consumed by mitochondria into O_2_^●−^ [19]. Specifically, in the mitochondrial matrix, complexes I and III transform oxygen into O_2_^●−^. Complex III in the intermembrane space of the mitochondria also produces O_2_^●−^. The activities of complexes in the ETC are diminished as a result of mitochondrial dysfunction caused by senescence [82, 83]. In particular, the lower activity of complex I, prevents it from performing effective electron transport, which leads to an increase in electron leakage to oxygen. This, in turn, increases the formation of O_2_^●−^ [84]. Enhanced ROS production in mitochondria exacerbates damage to the ETC, resulting in increased mitochondrial ROS generation [5]. The structure and function of organelles deteriorate as a consequence of this harmful cycle, which ultimately leads to senescence [85]. This causal linkage emphasizes that reducing mitochondrial ROS production is a crucial strategy to reverse senescence [5]. Here, we identified a unique mechanism by which *M. officinalis* reduces mitochondrial ROS generation through restoring mitochondrial function. Increased MMP by *M. officinalis* extract demonstrates increased proton transport, suggesting the active electron transport within the ETC [86]. Efficient electron transport reduces electron leak in the ETC, thereby reducing the production of by–product ROS [87]. Moreover, we observed that *M. officinalis* extract diminished dependence on glycolysis as an intracellular energy source, indirectly suggesting that *M. officinalis* extract recovers mitochondrial function as an energy generation. Furthermore, restoration of mitochondrial function via *M. officinalis* extract was accompanied by recovery of senescence-associated phenotypes [88, 89]. Here, we propose that reducing ROS generation in mitochondria using *M. officinalis* extract may be a first step toward a treatment for aging.

Oxidative stress induced by ROS is a primary contributor to skin aging [90]. ROS damages collagen that constitutes the dermis and destroys the structural stability of the subcutaneous tissue [91]. ROS also damage cell structure by modifying the phospholipids and proteins that make up cell membranes [92]. The adverse effects of ROS on skin aging can be aggravated by the property that ROS are unstable with unpaired electrons, which steals electrons from surrounding molecules and causes oxidative chain reactions in surrounding tissues. The adverse effects of ROS on skin aging can be exacerbated by the unstable nature of ROS, which has an unpaired electron [84]. This allows them to steal electrons from surrounding molecules, causing an oxidative chain reaction in surrounding tissues [84]. Thus, ROS damages large areas of the skin and further aggravates skin aging [80]. In this study, we elucidated the mechanism by which *M. officinalis* extract and its active ingredient, honokiol, reduce mitochondrial ROS generation. Specifically, we found that *M. officinalis* extract restores skin barrier formation by increasing skin regeneration and suppressing skin inflammation. Upregulation of genes involved in skin regeneration and downregulation of genes involved in collagen degradation indicated that *M. officinalis* extract restored the skin barrier function. Downregulation of genes involved in skin inflammation indicated that *M. officinalis* extract inhibit skin inflammation. *M. officinalis* extract also reversed skin aging by inhibiting skin pigmentation, increasing skin regeneration, and maintaining skin moisture. As far as we are aware, our research offers the first proof that *M. officinalis* and honokiol restore skin aging though reducing mitochondrial ROS generation. We propose that *M. officinalis* extract or honokiol alone may be effective in delaying or stopping skin aging, but further studies are needed to verify this suggestion.

Natural products are widely used in medicines, cosmetics, and health supplements because their side effects on the human body are minimized [93]. Since natural products are composed of various ingredients, it is important to identify the active ingredients. Finding active ingredients in natural products can help reduce the use of harmful or unnecessary additives [94]. Moreover, the composition of natural products may alter as a result of climate or farming area changes [95]. The effectiveness of products using natural products may also be affected by changes in the content [93, 96]. In this study, we investigated the components showing antioxidant activity among the known active ingredients of *M. officinalis*, honokiol and magnolol. Honokiol and magnolol are polyphenol compounds belonging to the lignan family [97]. Honokiol has a 5,5’-diallyl-2,2’-biphenol configuration and contains a biphenyl structure with two phenol rings connected by an allylic group attached to each ring [98]. Similarly, magnolol has an isomeric configuration featuring two phenol rings linked by a 5,5’-diallyl-2,2’-biphenol framework [99]. Phenolic compounds exhibit antioxidant properties through their hydroxyl groups [100, 101]. Specifically, the hydroxyl group of the phenol moiety exerts its antioxidant activity by acting as a singlet oxygen scavenger through redox reactions [102]. In this study, honokiol significantly reduced ROS levels in senescent fibroblasts at concentrations of 1 μM and 10 μM, and more than *M. officinalis*. However, magnolol reduced ROS levels at concentrations of 1 μM and 10 μM, but not more than *M. officinalis*. This difference is attributed to the structural differences between honokiol and magnolol [103]. The di-ortho-hydroxyl group of magnolol prevents the donation of hydrogen atoms to radicals by forming intramolecular hydrogen bonds [104]. In addition, magnolol has two hydroxyl groups at the -ortho position, whereas honokiol has an asymmetric structure with one hydroxyl group at the -ortho and -para positions, respectively [105]. This asymmetry of honokiol leads to an uneven electron distribution, making it prone to donating hydrogen atoms to ROS [106]. These structural characteristics support the conclusion that honokiol reduces ROS levels more efficiently in senescent fibroblasts. Furthermore, honokiol alone significantly reduced ROS levels in senescent fibroblasts compared to *M. officinalis* extract, indicating that honokiol is the most potent antioxidant among the components of *M. officinalis* extract.

In this study, magnolol was not selected as the main antioxidant component of *M. officinalis* extract because it was not as effective as honokiol in reducing mitochondrial ROS in senescent fibroblasts. However, another study reported the antioxidant effect of magnolol in nematodes [107]. Specifically, magnolol improved stress resistance to hydrogen peroxide, paraquat, and potassium mercury chloride in nematodes. The improved stress resistance led to a decrease in ROS in nematodes. The difference in the ROS-reducing effect of magnolol in senescent fibroblasts and nematodes may be due to the different levels of ROS depending on the level of aging. The ROS level of senescent fibroblasts was more than 10 times higher than that of young fibroblasts (Figure 1A). The ROS level of aged nematodes was also higher than that of juvenile nematodes [108]. This study used senescent fibroblasts with high ROS levels, whereas the nematodes used in the lifespan study were juvenile [107]. Therefore, the ROS-reducing effect of magnolol may have been more evident in young nematodes with relatively low ROS levels, but may not have been evident in senescent fibroblasts with high ROS levels. However, we acknowledge that further experiments are needed to verify this hypothesis.

Although there are previous studies on the antioxidant effects of *M. officinalis* extract, our study has some differences and advantages. First, other studies have demonstrated that *M. officinalis* reduces ROS levels using cells from colon adenocarcinoma, primary cells, squamous cell carcinoma, and normal gastric mucosa [109–112]. However, the effect of *M. officinalis* on reducing ROS levels in senescent fibroblasts has not been investigated. Our study is the first to confirm the effect of *M. officinalis* on reducing ROS in senescent fibroblasts. Second, our study made a novel finding that *M. officinalis* reduces mitochondrial ROS generation. To evaluate mitochondrial ROS levels, DHR123 and MitoSOX were used. Rhodamine 123, a dye that passively diffuses across cell membranes and selectively stains mitochondria, is reduced to form DHR123 [20]. Thus, DHR123 reacts with ROS in mitochondria and is oxidized to cationic rhodamine 123 [21]. Moreover, the lipophilic and positively charged TPP^+^ present in MitoSOX passes through the membrane and accumulates in the negatively charged mitochondrial matrix, where the dihydroethidium bound to TPP^+^ reacts with O_2_^●−^ present in the mitochondrial matrix [22]. However, other studies have used 2′,7′-dichlorofluorescein diacetate (DCF-DA) [109, 112], expression of inducible nitric oxide synthase (iNOS) [110] or Seahorse metabolic analysis [111]. DCF-DA can detect hydrogen peroxide, a type of ROS in the cytoplasm, because they are oxidized by hydrogen peroxide present in the cytoplasm, but they cannot specifically detect mitochondrial ROS generation [113, 114]. Moreover, iNOS is considered a pro-inflammatory marker and is not a direct method to measure ROS levels [115]. Furthermore, seahorse metabolic analysis is not a direct method for measuring ROS levels, but rather measures oxygen consumption and extracellular acidification rates [116]. Therefore, our study is the first to identify the effect of *M. officinalis* on reducing mitochondrial ROS generation in senescent fibroblasts.

In conclusion, we discovered that *M. officinalis* extract reduced ROS levels through mitochondrial functional recovery. Reduction in ROS levels by *M. officinalis* extract improved senescence-associated phenotypes and skin aging. In addition, honokiol, one of the components of *M. officinalis* extract, was found to be an effective substance with antioxidant activity. Honokiol also restored mitochondrial function, reducing ROS levels and alleviating aging-related phenotypes. Our findings reveal a unique mechanism by which *M. officinalis* extract reverses senescence. If further research is conducted to apply the new mechanism discovered in this study to clinical or cosmetic applications, it will be a turning point in the development of anti-aging treatments and cosmetics.

## MATERIALS AND METHODS

### Cell culture

All human cell line studies were reviewed and approved by the Animal Care and Use Committee of Incheon National University (protocol number: 20230115001). Immortalized human keratinocytes (HaCaT; 300493; Cytion, Eppelheim, Germany), human dermal fibroblasts (PCS–201–010; ATCC, Manassas, VA, USA), and normal human epidermal keratinocytes (HEKn; C0055C; Gibco, Grand Island, NY, USA) were employed. Each cell was cultured under specific media and culture conditions according to the procedures of previous studies [117]. Human dermal fibroblasts were classified into senescent and young fibroblasts based on their doubling time. Senescent fibroblasts were determined to have a doubling time of 14 days, while young fibroblasts were determined to have a doubling time of less than 2 days.

### Preparation of extract powder

The bark of *M. officinalis* (Pure Mind, Yeongcheon, Republic of Korea) was mixed with 70% ethanol in a volume ratio of 1:8 and heated at 60° C for 3 h. The roots of *Polygonum odoratum* (Hadong Agricultural Cooperative, Hadong, Republic of Korea) were mixed with 30% ethanol in a volume ratio of 1:10 and heated at 25° C for 3 h. The roots of *Magnolia Liliiflora* (*M. Liliiflora*) (Herb Village Co., Ltd., Cheongju, Republic of Korea) were mixed with deionized water in a volume ratio of 1:25 and heated at 60° C for 6 h. The roots of *Passiflora caerulea* (*P. caerulea*) (Jeju Plant Resource Research Institute, Jeju, Republic of Korea) were mixed with 70% ethanol in a volume ratio of 1:10 and heated at 60° C for 3 h. The extract powder was prepared according to the method used in a previous study [117]. Each extract was diluted with dimethyl sulfoxide (DMSO, D8418; Sigma, St. Louis, MO, USA) to a concentration of 100 mg/ml. 1 μl of 100 mg/ml extract was combined with 10 ml of medium to create a concentration of 10 μg/ml extract. By diluting DMSO in the medium to a concentration of 0.01%, DMSO control was employed. In particular, for DMSO control, 10 ml of medium was mixed with 1 μl of DMSO.

### Flow cytometric analysis of reactive oxygen species (ROS)

Senescent fibroblasts were treated with DMSO (0.01%), *M. officinalis* (10 μg/ml), *P. odoratum* (10 μg/ml), *M. Liliiflora* (10 μg/ml), or *P. caerulea* extract (10 μg/ml) for 12 days. As a positive control, 100 μM resveratrol (76511; Sigma) and young fibroblasts were used. Then, flow cytometric analysis of ROS was performed as in our previous study [117].

### Cellular proliferation assay

In 96-well plates (353072; BD Biosciences, Franklin Lakes, NJ, USA), senescent fibroblasts were seeded at a density of 1 × 10^3^ cells per well. Senescent fibroblasts were then treated with DMSO (0.01%) or *M. officinalis* extract (0.625, 1.25, 2.5, 5, or 10 μg/ml) for 12 days. Cell proliferation was calculated using a DNA content assay following the procedure of the previous study [117].

### Measurement of cell viability

Cell viability was measured once every 4 days for 12 days by the Cedex HiRes Analyzer (05650216001; Roche, Basel, Switzerland).

### Flow cytometric analysis of mitochondrial membrane potential (MMP), mitochondrial mass and autofluorescence

For 12 days, DMSO (0.01%) or *M. officinalis* extract (0.625 μg/ml) were used to treat senescent fibroblasts. Then, to evaluate MMP, senescent fibroblasts were incubated for 30 min at 37° C in medium with 0.6 μg/ml JC–10 (ENZ–52305; Enzo Life Sciences, Farmingdale, NY, USA). To assess mitochondrial mass, senescent fibroblasts were incubated for 30 min at 37° C in medium containing 50 nM MitoTracker Deep Red (M22426; Thermo Fisher Scientific, Waltham, MA, USA). To evaluate autofluorescence, senescent fibroblasts were incubated in dye-free medium for 30 min at 37° C. Following the protocol of the earlier investigation, flow cytometry analysis was then carried out [44].

### Measurement of extracellular acidification rate (ECAR)

The Seahorse XF Glycolytic Rate Assay Kit (103344–100; Agilent Technology, Santa Clara, CA, USA) was used to measure ECAR. The Seahorse XFe96 analyzer (Agilent Technology) was used in accordance with the manufacturer’s instructions.

### Senescent associated–β–galactosidase (SA–β–gal) staining

The manufacturer’s instructions for SA–β–gal staining (9860; Cell Signaling Technology, Beverly, MA, USA) were followed.

### Neutral comet assay

A CometAssay Single Cell Gel Electrophoresis Assay Kit (4250–050–K; R&D systems, Minneapolis, MN, USA) was used to measure the length of the DNA tail. The manufacturer’s procedure was followed.

### Quantitative polymerase chain reaction (qPCR)

qPCR was carried out as previously mentioned [118]. qPCR was done using the following primer (Table 1).

**Table 1:** Details of primers used in qPCR.

**Target**	**Orientation**	**Sequence (5′–3′)**	**Size (bp)**
*36B4*	forward	CAGCAAGTGGGAAGGTGTAATCC	23
	reverse	CCCATTCTATCATCAACGGGTACAA	25
*GAPDH*	forward	CAATGACCCCTTCATTGACC	20
	reverse	AAATGAGCCCCAGCCTTCT	19
*β–actin*	forward	GGCACCCAGCACAATGAAG	19
	reverse	CCGATCCACACGGAGTACTTG	21
*SLIT2*	forward	CAGAGCTTCAGCAACATGACCC	22
	reverse	GAAAGCACCTTCAGGCACAACAG	23
*COL1A1*	forward	AGCAAGAACCCCAAGGACAA	20
	reverse	CGAACTGGAATCCATCGGTC	20
*MMP1*	forward	ATGAAGCAGCCCAGATGTGGAG	22
	reverse	TGGTCCACATCTGCTCTTGGCA	22
*CCL2*	forward	AGAATCACCAGCAGCAAGTGTCC	23
	reverse	TCCTGAACCCACTTCTGCTTGG	22
*CCL5*	forward	CCTGCTGCTTTGCCTACATTGC	22
	reverse	ACACACTTGGCGGTTCTTTCGG	22
*IL–6*	forward	AGACAGCCACTCACCTCTTCAG	22
	reverse	TTCTGCCAGTGCCTCTTTGCTG	22
*IL–8*	forward	GAGAGTGATTGAGAGTGGACCAC	23
	reverse	CACAACCCTCTGCACCCAGTTT	22
*PAR–2*	forward	TGCTAGCAGCCTCTCTCTCC	20
	reverse	CCAGTGAGGACAGATGCAGA	20
*SDF–1*	forward	TGCCAGAGCCAACGTCAAG	19
	reverse	CAGCCGGGCTACAATCTGAA	20
*Bmal–1*	forward	TGTGGGCGCTCACTGTGT	18
	reverse	TTCTGCCTGATCCTGTCATCTCT	23

### Measurement of melanosome transport

In order to promote the development of melanosomes, HaCaT cells were exposed to 30 mJ/cm^2^ UVB. Next, DMSO (0.01%) or *M. officinalis* extract (0.625, 1.25, and 2.5 μg/ml) were applied to HaCaT cells for 8 h. Niacinamide (N0636; Sigma) at a concentration of 100 μg/ml was employed as a positive control. Deionized water was used to dilute niacinamide to a concentration of 1 g/ml. To reach 100 μg/ml, 10 ml of medium was mixed with 1 μl of 1 g/ml niacinamide.

### Measurement of skin pigmentation

In order to cause skin pigmentation, 15 J/cm_2_ UVB was applied to HaCaT cells. Next, DMSO (0.01%) or *M. officinalis* extract (0.625, 1.25, and 2.5 μg/ml) were applied to HaCaT cells for a duration of 24 h. 1 μM retinol (R7632; Sigma) was employed as a positive control. DMSO (D8418; Sigma) was used to dilute the retinol to a concentration of 10 μM. To reach a concentration of 1 μM, 10 ml of medium was mixed with 1 μl of 10 μM retinol.

### Measurement of skin turnover

30 mJ/cm_2_ UVB was applied to HaCaT cells in order to interfere with the skin turnover cycle. Next, DMSO (0.01%) or *M. officinalis* extract (0.625, 1.25, and 2.5 μg/ml) were applied to HaCaT cells for a duration of 24 h.

### Western blot analysis

The Western blot analysis procedure was carried out as previously mentioned [119]. This study employed the following antibodies: horseradish peroxidase (HRP)-conjugated secondary antibody (1706515; Bio-Rad, Hercules, CA, USA, 1:2,000 dilution in 5% skim milk), HRP-conjugated secondary antibody (1706516; Bio-Rad, 1:10,000 dilution in 5% skim milk), PAR–2 antibody (sc-13504; Santa Cruz biotechnology, Dallas, TX, USA, 1:500 dilution in 5% skim milk), GAPDH antibody (sc-32233; Santa Cruz biotechnology, 1:5,000 dilution in 5% skim milk), SDF–1 antibody (ab155090; Abcam, Cambridge, UK, 1:1,000 dilution in 5% skim milk), and Bmal–1 antibody (PA1-523; Invitrogen, Waltham, MA, USA, 1:1,000 dilution in 5% skim milk).

### Measurement of skin moisture retention

HEKn cells, which are normal human epidermal keratinocytes, were exposed to DMSO (0.01%) or *M. officinalis* extract (0.625, 1.25, and 2.5 μg/ml) for a duration of 72 h. HEKn cells were treated with 250 μg/ml ceramide NP (100403-19-8; Evonik, Germany) as a positive control. DMSO (D8418; Sigma) was used to dilute ceramide NP to a concentration of 2.5 g/ml. 1 μl of 2.50 g/ml ceramide NP was added to 10 ml of medium to reach a concentration of 250 μg/ml. Alexa Fluor® 488 goat anti-rabbit IgG antibody (1:400 dilution; A28175; Invitrogen) was the secondary antibody utilized, whereas the primary antibody was anti-Filaggrin (1:200 dilution; PA5–83128; Invitrogen). To measure the fluorescence intensity of the filaggrin protein, Image J analysis (National Institutes of Health) was used.

### Statistical analysis

A standard statistical software package (GraphPad Prism 9; San Diego, CA, USA) was used to perform the statistical analyses. To ascertain if differences were significant, the student’s t–test, two–way ANOVA, and Bonferroni’s post–hoc test were employed.

### Data availability statement

The original contributions presented in the study are included in the article, further inquiries can be directed to the corresponding authors.

## Supplementary Material

Supplementary Figure 1
