# Supplementary material for: Identification of senescence rejuvenation mechanism of Magnolia officinalis extract including honokiol as a core ingredient
Source: Aging (Albany NY). 2025 Feb 21;17(2):497–523. doi: 10.18632/aging.206207 (PMC11892931; doi:10.18632/aging.206207)
Supplement: Supplementary Figure 1 [file aging-17-206207-s001.pdf]

SUPPLEMENTARY FIGURE

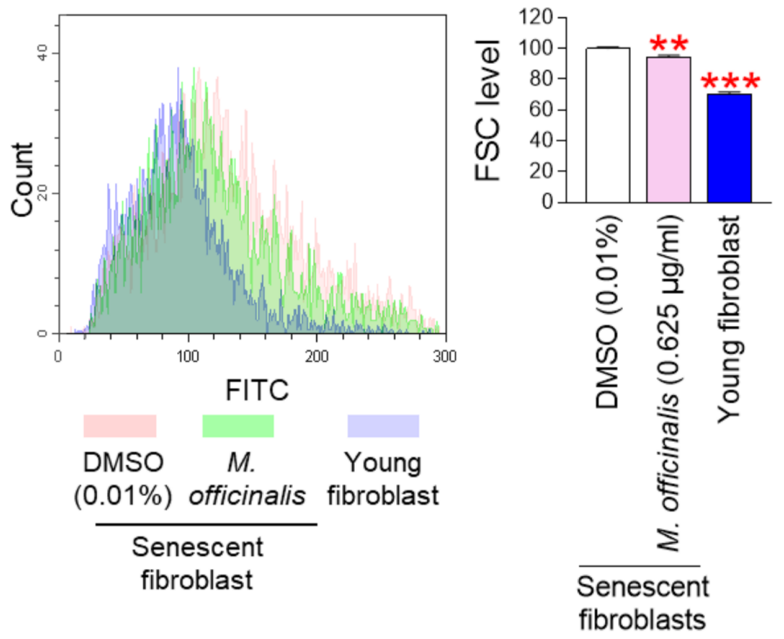

Supplementary Figure 1. Flow cytometry analysis of forward scatter (FSC). After 12 days of treatment with DMSO (0.01%) or honokiol (1 µM), FSC level was by flow cytometry. The representative histogram of FSC was shown. **$P < 0.01$, ***$P < 0.001$, Student t–test. Mean ± S.D., N = 3.
